# Investigation of the Effects of Roller Spreading Parameters on Powder Bed Quality in Selective Laser Sintering

**DOI:** 10.3390/ma15113849

**Published:** 2022-05-27

**Authors:** Xiangwu Xiao, Yufeng Jin, Yuanqiang Tan, Wei Gao, Shengqiang Jiang, Sisi Liu, Meiliang Chen

**Affiliations:** 1 School of Mechanical Engineering, Xiangtan University, Xiangtan 411105, China; 13669902083@163.com (Y.J.); jsqcx@126.com (S.J.); liusisi@xtu.edu.cn (S.L.); 2 Institute of Manufacturing Engineering, Huaqiao University, Xiamen 361021, China; tanyq@hqu.edu.cn; 3 School of Electro-Mechanical Engineering, Guangdong University of Technology, Guangzhou 510006, China; gaowei@gdut.edu.cn

**Keywords:** selective laser sintering, spread the powder quality, parameter optimization, DEM, RSM, NSGA-II

## Abstract

Powder spreading is one of crucial steps in selective laser sintering (SLS), which controls the quality of the powder bed and affects the quality of the printed parts. It is not advisable to use empirical methods or trial-and-error methods that consume lots of manpower and material resources to match the powder property parameters and powder laying process parameters. In this paper, powder spreading in realistic SLS settings was simulated using a discrete element method (DEM) to investigate the effects of the powder’s physical properties and operating conditions on the bed quality, characterized by the density characteristics, density uniformity, and flatness of the powder layer. A regression model of the powdering quality was established based on the response surface methodology (RSM). The relationship between the proposed powdering quality index and the research variables was well expressed. An improved multi-objective optimization algorithm of the non-dominated sorting genetic algorithm II (NSGA-II) was used to optimize the powder laying quality of nylon powder in the SLS process. We provided different optimization schemes according to the different process requirements. The reliability of the multi-objective optimization results for powdering quality was verified via experiments.

## 1. Introduction

Selective laser sintering (SLS) is one of the typical additive manufacturing processes, which creates objects via scanning and layer-by-layer sintering. As a novel technology used for the design and manufacturing of complex shapes and structures, SLS is implemented a fast rate for automobile, shipbuilding, aerospace, and medical applications [1,2]. The laying of a flat, uniform, and high-density powder bed is the aim when preparing the molded parts to ensure good performance [3,4]. The size accuracy and mechanical properties of the sintered parts are directly affected by the powder laying quality, which is closely related to the powder flow characteristics and powder laying process parameters [5].

Flowability is an essential powder property for the achievement of uniformly spread powder layers [6]. The powder must have appropriate rheological properties to form thin, dense, and uniform powder layers [7]. The commonly used characterization methods for powder fluidity include the angle of repose method, outflow velocity method, Hausner index method, Carr fluidity index method, and shear method [8,9]. The powder flow characteristics depend on many parameters, such as the particle size distribution [10,11], particle shape [12,13], interparticle interaction force [14], and temperature [15]. For example, Wei et al. [16]’s research suggests that the surface shape affects the stability of the particle stacking structure and the uniformity of the pore distribution. Dai et al.’s research showed that [14] both the sliding friction and rolling friction hinder the particle flowability, leading to a higher angle of repose and a lower packing fraction in the sandpile. 

The technological parameters of the roller spreading process are relatively complex, which include the thickness of the powder layer and the diameter, rotation speed, and displacement speed of drum [17]. It is not advisable to optimize the powder laying process through experience and tedious experiments. Therefore, it is necessary to optimize the technological parameters of the roller powder laying process via numerical simulation to improve the spreading properties of the powder. The discrete element method (DEM) has great advantages in simulating the motion of powder systems [18,19]. The basic idea of the DEM is to divide the system into a number of particles, whereby the response of the whole system is described through the mechanical and kinetic states of each particle in the system [20]. The DEM has been widely used to investigate the flow mode and dynamic behavior of powder particles in additive manufacturing and to reveal the effects of the powder laying process on the powder laying quality [21,22]. For instance, Meier [23] studied the influence of the particle size distribution and adhesion forces between particles on the uniformity of the powder layer in additive manufacturing. Tan et al. [24] established a contact model between powder particles, which took van der Waals forces between particles into account. The parameters of the contact model were calibrated experimentally. The powder laying process was simulated, the density uniformity of powder layer was evaluated, and the fluidity of the new powder and residual powder was compared. 

It is of great significance to establish the relationship between powder property parameters, powder laying process parameters, and powder laying quality to expand the raw material range of the powder promotion process. The evaluation index of the powder spreading quality can be divided into powder quality (such as the powder density, powder spreading thickness, coverage rate, and surface uniformity) and powder flow morphology (such as deposition rate and avalanche angle change rate) aspects. More scholars are focusing on the influence of the powder laying process on the powder laying quality. Mussatto et al. [7] systematically studied the effects of the powder morphology, diffusion rate, and layer thickness on the powder bed morphology uniformity. Chen [25] studied the fluidity and powder quality of the powder laying process. The results showed that the continuity and stability of the powder flow decrease with the increase in powder spreading speed and the decrease in powder spreading layer thickness, which lead to the deterioration of the bulk density and uniformity. Yao et al. [26] simulated the powder laying process with a 316L stainless steel powder scraper. The effects of technological parameters, the scraper structure, and the powder particle size on the powder laying quality were studied. The optimum process parameters were determined. Parteli et al. [27] developed a DEM numerical tool for the SLS powder laying process, with which the characteristics of the powder layer deposited on the parts are studied by applying it to the roller powder dispensing system. The results showed that an increase in powder spreading speed and wider particle size distribution will lead to an increase in the surface roughness of the powder layer, and will ultimately affect the quality of the parts.

The powder laying process parameters and physical powder parameters affect each other and affect the quality of the powder laying process. At present, some researchers still use empirical or trial-and-error methods in this process, which consume more manpower and material resources to match the powder property parameters and powder laying process parameters. Although DEM simulation of the SLS powder laying process can monitor the powder laying quality well, this approach requires a lot of time because the powder size is very small, the simulation system is huge, and the computing capacity is limited. In the development of various optimization methods, the response surface methodology (RSM) and genetic algorithm (GA) are used to optimize parameters to solve engineering problems [28,29,30]. The multi-objective optimization method, which uses polynomials to fit the relationship between factors and responses, can simplify these engineering problems. The influences of the single factor and interaction factor on the response index were analyzed previously and the optimal parameters were obtained [31]. 

In this paper, powder spreading in realistic SLS settings was simulated using the DEM to investigate the effects of the powder’s physical properties and operating conditions on the bed quality, characterized by its density characteristics, density uniformity, and flatness of the powder layer. The central composite design (CCD) approach was used to generate 13 groups of cases and to establish the regression model of the 3 indicators. A regression model of the powdering quality was established based on the response surface methodology. According to the analysis of variance (ANOVA), the influences of single factors and their interactions on the response indicators were determined. Multi-objective optimization was carried out for the drum powder laying parameters and the optimization results were verified via experiments. This study will be helpful to optimize the drum powder laying process parameters and improve the powder laying quality in the SLS process.

## 2. Methods

### 2.1. Discrete Element Method

In this model, based on the Hertz–Mindlin model and SLS powder paving process, the particle gravity, collision force between particles (between particles and wall), friction, van der Waals force, and electrostatic force were comprehensively considered to describe the contact dynamic behavior of nylon powder at preheating temperature via DEM. There are two modes of motion, namely translational motion and rotational motion, which describe the motion of particles according to Newton’s second law of motion:

(1) $$m_{i}\frac{dv_{i}}{dt}=\sum_{j}F_{ij}^{c}+\sum_{k}F_{ik}^{nc}+F_{i}^{g}$$

(2) $$I_{i}\frac{d\omega_{i}}{dt}=\sum_{j}M_{ij}$$

where $F_{ij}^{e}$ is the contact force of particle j to particle i or wall j to particle i, $F_{ik}^{nc}$ is the non-contact force of particle k to particle i or wall k to particle i, and $F_{i}^{g}$ is the self-gravity of nylon power i; $v_{i}$ is the position vector of the particle i, $\omega_{i}$ is the angle vector of the particle i, and $M_{ij}$ is the torque of particle j to particle i or wall j to particle i.

$F_{ij}^{c}$ can be decomposed into the normal contact force $F_{nc}$ and tangential contact force $F_{tc}$. The contact force $F_{nc}$ of nylon powder i in the normal direction is composed of the normal elastic force $F_{nc,s}$, normal damping force $F_{nc,d}$,van der Waals $F_{nc}^{vdw}$, and static force $F_{nc}^{ele}$:

(3) $$F_{nc}=F_{nc,s}+F_{nc,d}+F_{nc}^{vdw}+F_{nc}^{ele}$$

The Van der Waals forces take into account only the gravitational component based on Hmaker’s theory. The normal elastic force $F_{nc,s}$ and normal damping force $F_{nc,d}$ can be obtained according to the Hertz–Mindlin model:

(4) $$F_{n,s}^{c}=\frac{4}{3}E^{*}\sqrt{R^{*}}\delta_{n}{}^{3/2}$$

(5) $$F_{n,d}^{c}=-2\sqrt{\frac{5}{6}}\beta \sqrt{S_{n}m^{*}}v_{n}^{\bar{rel}}$$

where $E^{*}$ is the equivalent elastic modulus of nylon powder, $R^{*}$ is the equivalent radius of nylon powder, $m^{*}$ is the equivalent mass of nylon powder, β is the damping coefficient, and $S_{n}$ is the normal contact stiffness, the expression of which is as follows:

(6) $$E^{*}=\frac{E_{p}}{2(1-\gamma_{p}^{2})}$$

(7) $$R^{*}={\left[\frac{1}{R_{i}}+\frac{1}{R_{j}}\right]}^{-1}$$

(8) $$m^{*}={\left[\frac{1}{m_{i}}+\frac{1}{m_{j}}\right]}^{-1}$$

(9) $$\beta =\;\frac{Ine}{\sqrt{In^{2}e+\pi^{2}}}$$

(10) $$S_{n}=2E^{*}\sqrt{R^{*}\delta_{n}}$$

where $E_{p}$ is the elastic modulus of nylon powder, $\gamma_{p}$ is Poisson’s ratio of nylon powder, and $m_{i}$ and $m_{j}$ are the mass of nylon powder i and j, respectively; e is the recovery coefficient of collision between nylon powders, $\delta_{n}$ is the normal overlap quantity, and $v_{n}^{\bar{rel}}$ is the normal relative velocity of contacting nylon powder.

Van der Waals forces are inherent in fine particle flows [32]. In the study of fine particle flow, the Hamaker theory is often used to calculate Van der Waals forces between fine particles [33]:

(11) $$F_{PP}^{vdw}=-\frac{\partial U_{PP}^{0}}{\partial Z_{0}}=-\frac{A_{pp}}{12Z_{0}^{2}}\frac{d_{i}d_{j}}{d_{i}+d_{j}}$$

(12) $$F_{pw}^{vdw}=-\frac{\partial U_{pw}^{0}}{\partial Z_{0}}=-\frac{A_{pw}d_{i}}{12Z_{0}^{2}}$$

where $F_{PP}^{vdw}$ is the Van der Waals force between particles, $F_{pw}^{vdw}$ is the Van der Waals force between particles and walls, $d_{i}$ and $d_{j}$ are the diameters of particles i and j, $Z_{0}$ is the distance between the particles, $A_{pp}$ is the Hamaker constant between powders, and $A_{pw}$ is the Hamaker constant of the powder and wall. 

The friction charge of the powder involves the friction charge between the powder and the wall, as well as between the powders. The electrostatic force between two charged particles is calculated by:

(13) $$F_{pp}^{ele}=\frac{1}{4\pi \varepsilon_{0}}\frac{q_{i}q_{j}}{r_{ij}^{2}}n_{ij}$$

where $q_{i}$ and $q_{j}$ are the charges of particles i and j, respectively; $r_{ij}$ is the distance between the centers of particle i and j, $\varepsilon_{0}$ is the dielectric constant of vacuum, and $n_{ij}$ is the unit vector from particle i to particle j.

The electrostatic force between the particle and the conducting plane is:

(14) $$F_{pw}^{ele}=\frac{1}{4\pi \varepsilon_{0}}\frac{q_{i}{}^{2}}{{\left[2(Z_{0}+s)\right]}^{2}}n_{pw}$$

where s is the correction factor and $n_{pw}$ is the unit vector. Refer to the literature for detailed information on static force modeling [34].

### 2.2. Establishment of Powder Laying Process Model

Nylon powder was selected as the research object in this study. The DEM model of the powder laying process established in this research is based on PA3200 powder. The preheating temperature of the SLS powder laying process is 171 °C. The contact parameters of nylon powder in DEM simulation are calculated according to the inverse parameter results. The DEM simulation results agree well with the experimental results. The reliability and accuracy of the DEM model at preheating temperature were verified. 

**Figure 1:**
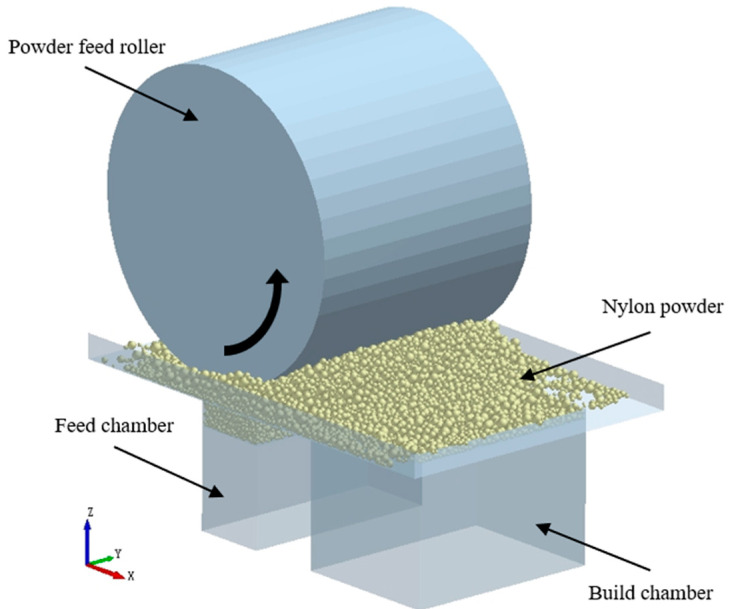
DEM simulation of roller spreading processes.

The above research laid a foundation for the study of the PA3200 SLS powder laying process. Figure 1 shows the SLS powder DEM model established in this study. The construction and verification process of the DEM model are detailed in our previous work [34,35]. Table 1 and Table 2 present the physical parameters and working parameters, respectively, in the SLS powder laying process.

### 2.3. Quality Index of Powder Laying

It is necessary to characterize the quality of nylon powder in the molding area before studying the influence of powder laying process parameters and powder property parameters on the quality of nylon powder laying in the molding area. In this study, the quality of powder laying is expressed by the density characteristics, density uniformity, and flatness of the powder layer in the formation area. 

**Figure 2:**
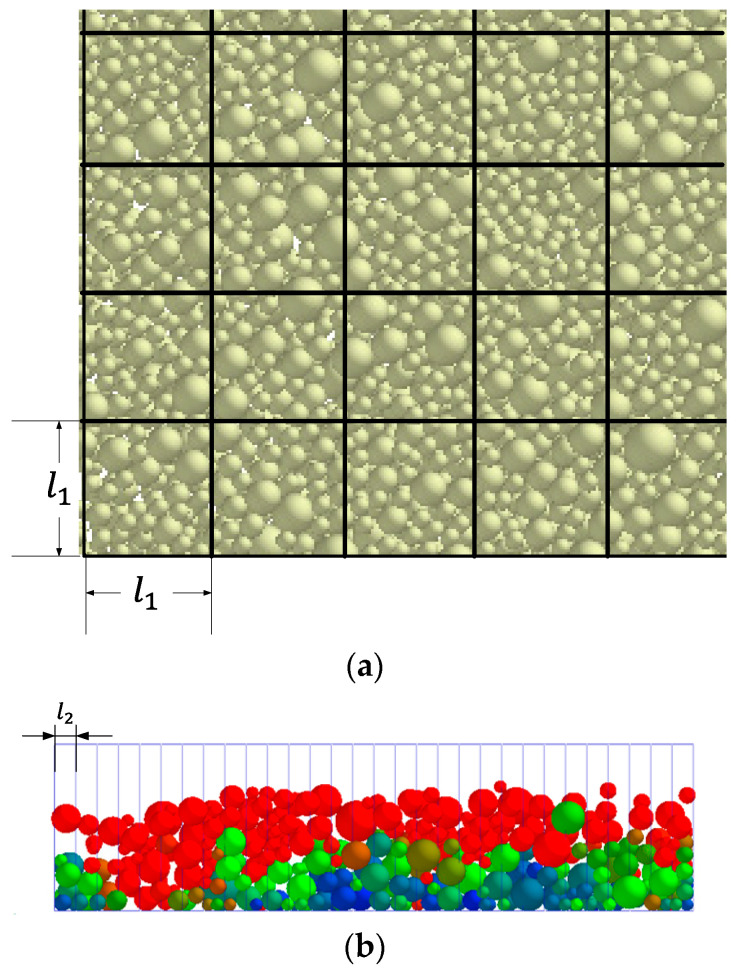
Grid division diagram of apparent density statistics: (**a**) meshing of horizontal plane of powder layer in formation area; (**b**) grid division of vertical plane of powder layer in formation area.

A schematic diagram of the area meshing used to measure the apparent density is shown in Figure 2. The density of the powder layer in the formation area is characterized by the ratio of the total particle mass to the particle volume of the layer:

(15) $$\rho =\frac{\sum_{i=1}^{n}m_{i}}{\sum_{i=1}^{n}v_{i}}$$

where $v_{i}$ is the volume of grid i and $m_{i}$ is the particle mass of grid i.

The standard deviation of the apparent density of the powder layer in the formation region is denoted by S, which can be used to represent the density uniformity of the powder layer. The standard deviation *S* of the apparent density of the powder layer can be expressed as:

(16) $$S=\sqrt{\frac{1}{N-1}\sum_{i=1}^{N}{\left(\rho_{i}^{\prime }-\bar{\rho^{\prime }}\right)}^{2}}$$

where $\bar{\rho^{\prime }}$ is the average apparent density of particles in the selected box, which is given by:

(17) $$\bar{\rho^{\prime }}=\frac{\sum_{i=1}^{N}\rho_{i}^{\prime }}{N}$$

Here, $R_{a}$ is the surface roughness. This can be used to characterize the flatness of the powder layer, which is given by [36]:

(18) $$R_{a}=\frac{1}{l}\int_{0}^{l}\left|y(x)\right|dx$$

where l is the sampling length, y(x) is the distance between the contour point and the reference line in the x direction, and the reference line is the least squares centerline of the contour.

**Figure 3:**
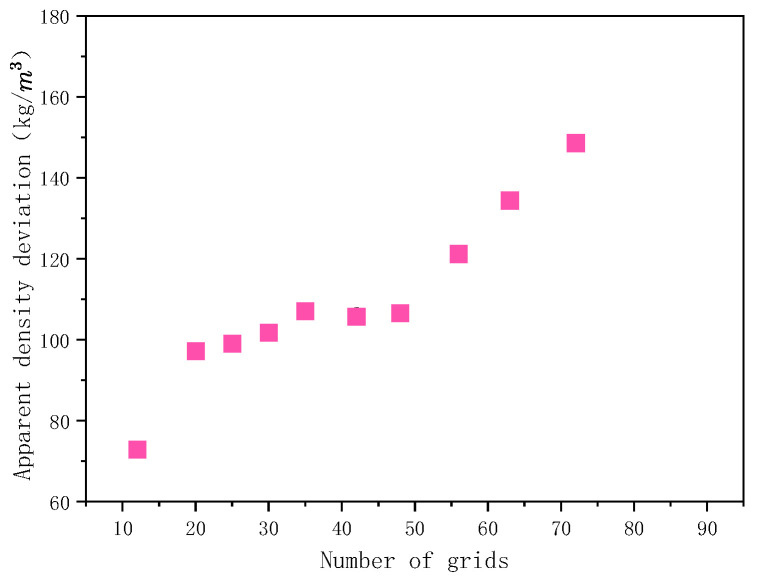
Influence of grid number on standard deviation of the density.

**Figure 4:**
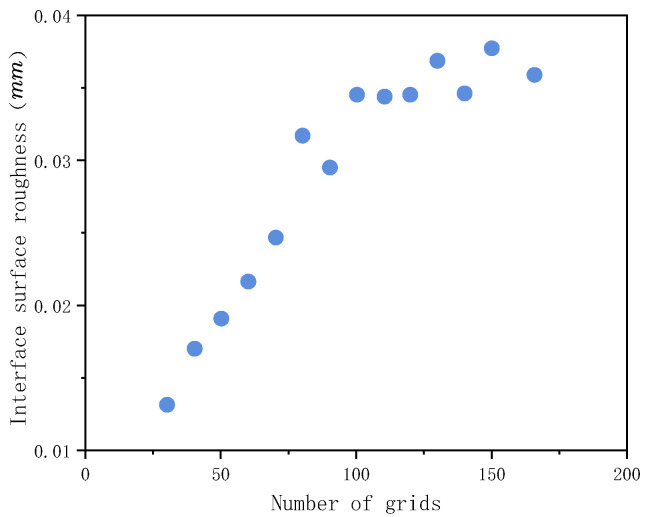
Effect of grid number on surface roughness.

In order to study the influence of the number of grids on the flatness of the powder layer in the formation area, the sampling lengths on the horizontal and vertical sections are divided by different number of grids. Figure 3 shows the effect of the mesh number on the standard deviation of the apparent density of powder layer $\bar{\rho^{\prime }}$ in the formation area. It can be seen that $\bar{\rho^{\prime }}$ increases with the increase in mesh number. When the number of grids increases from 20 to 48, the number of particles in each grid increases, but $\bar{\rho^{\prime }}$ is less sensitive to the number of grids. The effect of the number of grids on the surface roughness of the powdering layer $R_{a}$ in the formation area is shown in Figure 4. When the number of grids increases to a certain extent, $R_{a}$ does not change much. Therefore, the number of grids should not be too large when calculating the surface roughness of the powder layer in the formation area. In order to find out the optimum cell size, the mesh size of the powder layer selection box in the formation area should meet *S*_1_ ≥ 2.5*d_max_* and *S*_2_ ≤ 0.5*d_min_*. Here, *d_max_* is the maximum particle diameter and *d_min_* is the minimum particle diameter.

### 2.4. Response Surface Methodology

The response surface methodology (RSM) proposed by British statisticians Box and Wilson in 1951 [37] is frequently used to approximately fit unknown functions, such as the relationship between variables and responses. In practical applications, RSM establishes mathematical relations through regression analysis of the test results of physical experiments or simulation tests, which can evaluate the relevant factors and their interactions to determine the optimal level range. RSM has been successfully applied to optimize a variety of processes [38,39,40].

The basic idea of RSM can be summarized as follows:

(19) $$y=f\left(x_{1},x_{2},\cdots ,x_{p}\right)+\varepsilon$$

where *y* is a variable, $f\left(x_{1},x_{2},\cdots ,x_{p}\right)$ is the response function of factor $x_{1},x_{2},\cdots ,x_{p}$, and ε is the residual of the construction model.

The second-order response surface model is:

(20) $$y=\beta_{0}+\sum_{i=1}^{m}\beta_{i}x_{i}+\sum_{i=1}^{m}\beta_{ii}x_{i}^{2}+\sum_{i<j}^{m}\beta_{ij}x_{i}x_{j}+\varepsilon$$

where $\beta_{0}$ is the constant term, $\beta_{i}$ is the linear effect of $x_{i}$, $\beta_{ij}$ is the interaction effect of $x_{i}$ and $x_{j}$, and $\beta_{ii}$ is the second-order response of $x_{i}$. The second-order response surface method mainly includes the central composite design (CCD), Box–Behnken design, uniform design, and D-optimal design. The most commonly used second-order response surface design method is the central composite design method, which is used to optimize the reaction process parameters or to find the best synthesis conditions [41]. The central composite design method includes the universal rotary composite design, quadratic orthogonal composite design, and others. In this research, the universal rotating combination design is used to design the DEM simulation test scheme.

The general rotating composite design experiment was carried out considering the drum translation speed Vs (mm/s) and particle size d (mm) of D50 as experimental factors. The design factors of the DEM simulation test for the nylon powder laying process are shown in Table 3. The CCD model of RSM was used to generate 13 cases. The response indexes of the apparent density $Y_{1}$, standard deviation of the density $Y_{2}$, and powder layer roughness $Y_{3}$ can be calculated using Equation (18). Table 4 shows the simulation results of the corresponding indicators.

### 2.5. Multi-Objective Optimization Method Based on Genetic Algorithm

The multi-objective optimization problems (MOP) approach was first proposed by the Italian economist V. Pareto in 1896 [42]. The optimization objective can be expressed as:

(21) $$\begin{array}{c} \min\;\;Y=F\left(X\right)=\left[F_{1}\left(X\right),F_{2}\left(X\right),\cdots ,F_{m}\left(X\right)\right]\\ s.t.g_{i}\left(X\right)\leq 0\ldots \\ h_{j}\left(X\right)=0,j=1,2,\cdots ,q \end{array}$$

where X is the optimization parameter vector, $X=\left(x_{1},x_{2},\cdot \cdot \cdot ,x_{m}\right)\in D$, Y is the optimization target vector, and $Y=\left(f_{1},f_{2},\cdot \cdot \cdot f_{m}\right)\in F$.

In general, different objectives are in conflict with each other for MOP. There is no single optimal objective solution for MOP, but the Pareto optimal solution is set [43]. The Pareto optimal solution set is defined as ∀x∈Ω; if $x^{\prime }\in \Omega$ does not exist in the domain Δx, let $(x^{\prime }+\Delta x)\in \Omega$, when the following conditions are satisfied:

(22) $$F_{i}\left(x^{\prime }+\Delta x\right)\leq F_{i}\left(x^{\prime }\right)$$

(23) $$F_{j}\left(x^{\prime }+\Delta x\right)\leq F_{j}\left(x^{\prime }\right)$$

We note that $\;x^{\prime }\in \Omega \;$ is the Pareto optimal solution set used for multi-objective optimization.

The non-dominated genetic algorithm II (NSGA-II) is a kind of multi-objective genetic optimization algorithm, which was proposed by Kalyanmoy et al. in 2002 [44]. In this research, the NSGA-II improved algorithm Gamultiobj function provided by MATLAB is used to optimize the powder laying quality.

## 3. Results and Discussion

### 3.1. Variance Analysis and Regression Model Establishment

Design-expert 8.0.6 was used to conduct an RSM analysis on the DEM simulation results from the SLS powder laying process shown in Table 4. The response surface equation for the powdering quality can be obtained via regression analysis of the numerical simulation results. The apparent density, standard deviation of the density, and surface roughness formulae of the powder layer in the formation area are as follows:

(24) $$Y_{1}=716.3832-0.3129v_{s}-3.7645d+0.0008v_{s}{}^{2}+0.0234d^{2}$$

(25) $$Y_{2}=149.1053-0.33340v_{s}-2.0568d+0.0011v_{s}{}^{2}+0.0194d^{2}$$

(26) $$Y_{3}=57.6460-0.1181v_{s}-0.1330d+0.0006v_{s}d+0.0002dv_{s}{}^{2}$$

where $v_{s}$ is the translational speed of the drum and d is the diameter of particle D50.

Analysis of variance (ANOVA) is used to test the significance of the fitted second-order regression equation. The drum translation velocity *Vs* (mm/s) and the particle size D (mm) of the powder D50 are selected as independent variables of the multi-objective optimization model. In the optimization of powder laying process in the formation area, there are three objectives to be optimized, namely the maximum apparent density, the minimum standard deviation of the apparent density, and the minimum surface roughness. The regression model of the powdering quality established by the RSM is the objective function to be optimized, $F_{1}\left(x\right)=-Y_{1}$, $F_{2}\left(x\right)=-Y_{2}$, $F_{3}\left(x\right)=-Y_{3}$.

If the drum speed is too slow, the production efficiency will be affected; if the speed is too fast, the powder laying quality will be reduced. Therefore, the interval constraint is 100 ≤ *Vs* ≤ 300 (mm/s). The particle size of D50 is mainly controlled by the thickness of the powder layer, and the interval constraint is 50 ≤ D ≤ 100 (μm).

The crossover rate is 0.8, the population size is 100, the maximum evolution algebra is 200, the stop algebra is 200, and the deviation of the fitness function is $10^{-100}$. The variation rate is determined by the feasible region adaptation equation. The adaptive feasible mutation method can be used to assess the diversity of the population, which is conducive to the optimization of the results. The tolerance is set to $10^{-4}$ as the termination condition of the calculation. The other parameters are set to recommended values.

### 3.2. Effects of Powder Laying Process Parameters on Powder Laying Quality Index

**Figure 5:**
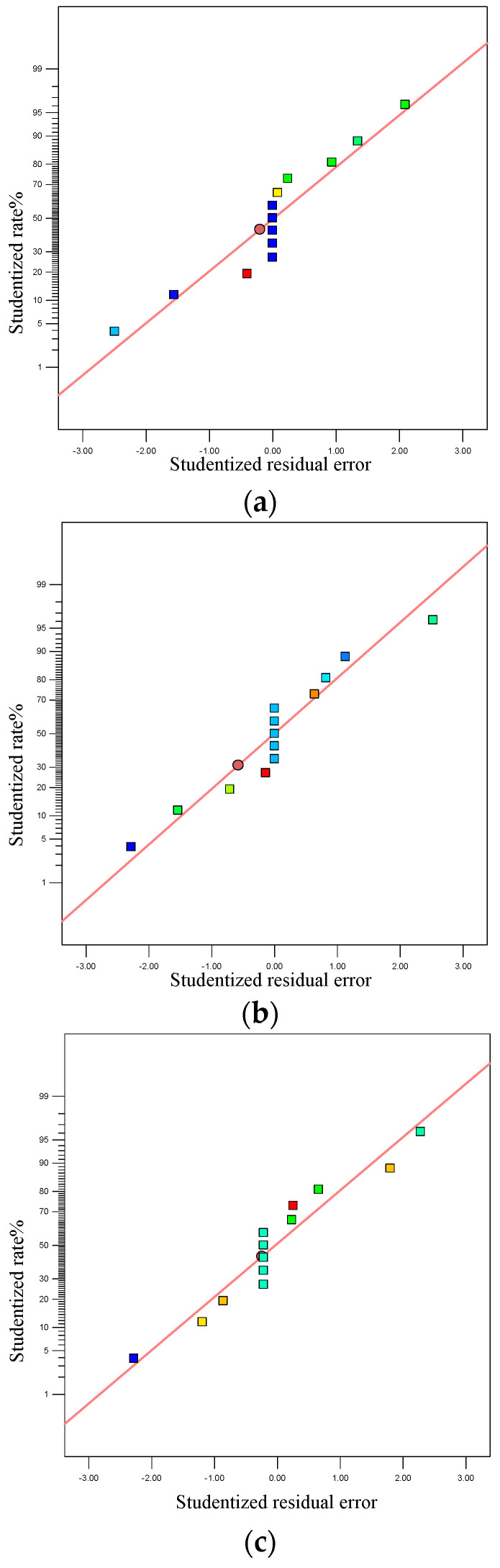
Normal residual diagram of powder quality from DEM simulation: (**a**) normal residual diagram of apparent density; (**b**) normal residuals of standard deviation of the density; (**c**) normal residual diagram of powder surface roughness.

In the formation area, the distribution of the normal residual diagram includes the apparent density of the powder layer, the standard deviation of the apparent density, and the surface roughness, as shown in Figure 5. It can be seen that the distribution of the residual points is almost in a straight line. The results show that the second-order model fitting effect of the nylon powder quality in the SLS process is good.

**Figure 6:**
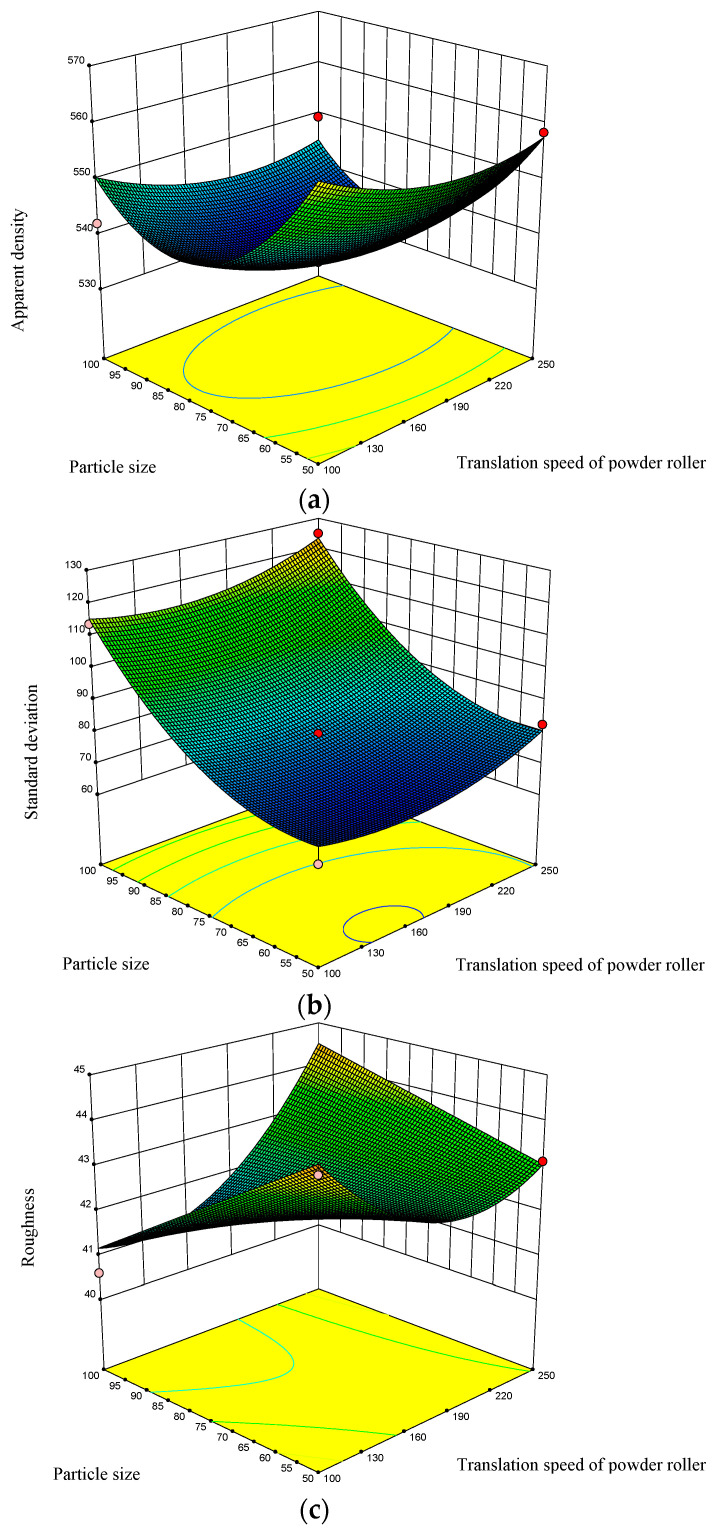
Response surface diagram of powdery mass from DEM simulation: (**a**) performance density; (**b**) standard deviation of the density; (**c**) roughness.

The response surface diagram of the relationships among the drum translational velocity, particle size, and powder laying quality is shown in Figure 6. Based on the response surface diagram, the influence of a single factor on the process parameters (drum translation speed) and powder property parameters (nylon powder particle size) can be assessed, and the synergistic influence of these parameters on the powder laying quality can be obtained.

The analysis shows that the particle size has a great influence on the apparent density, standard deviation of the density, and roughness of the powder layer in the formation area. The smaller the particles are, the more likely they are to agglomerate under the action of electrostatic and van der Waals forces. Therefore, the pores left by the roller powder are smaller and the densification degree of the powder bed is also increased. The smaller particle size improves the apparent density of the powder layer, reduces the standard deviation of the density, and improves the density uniformity, but is not conducive to reducing the surface roughness. The effects of the roller translation speed on the apparent density and density uniformity of the formation area are relatively small, but the effect on the roughness is greater. With the increase in drum translation speed, the apparent density of the powder layer in the formation area decreases slowly. When the drum translation speed increases to a certain extent, the apparent density of the powder layer will increase slightly, although the overall change trend will be small. However, this is contrary to the effects of the roller translational velocity on the density uniformity and roughness. If the roller translation speed is too high or too low, this will not be conducive to improving the uniformity of the powder layer density and reducing the roughness of the powder layer surface. The apparent density of the powder layer is in conflict with the standard deviation of the apparent density and the surface roughness in the target formation area. This is also consistent with the previous simulation results. The regression equation for the powdering quality established based on the RSM is reliable and can predict the powdering quality well.

### 3.3. Multi-Objective Optimization Results for the Powder Laying Quality

Based on the Gamultiobj function, 80 Pareto optimal solutions were obtained to assess the nylon powder quality during the SLS process. Partial Pareto optimal solutions are given in Table 5. Here, an optimal compromise solution is selected in the Pareto set according to product preference. The first solution is biased towards the maximum apparent density of the powder layer in the formation region. The second solution is biased towards the best uniformity of the powder layer density in the formation region. The third solution is biased towards the optimal surface flatness of the powder layer in the formation region. If all three are considered, the fourth solution can be selected as the optimal compromise solution. When the particle size of the powder is determined in the actual engineering process, the appropriate drum translation speed can be selected according to Table 5 to optimize the powder laying quality. Through this method, the matching of the physical property parameters and the SLS powder laying process parameters and the prediction of powder laying quality were achieved.

## 4. Experimental Verification

PA3200 powder with a particle size of 50.02 mm was selected as the experimental material, The polymer powder sintering machine (FS251) designed and manufactured by Hunan Hua Shu Hi-tech co., Ltd., was used for the powder laying experiment. The molding process parameters are shown in Table 6.

**Figure 7:**
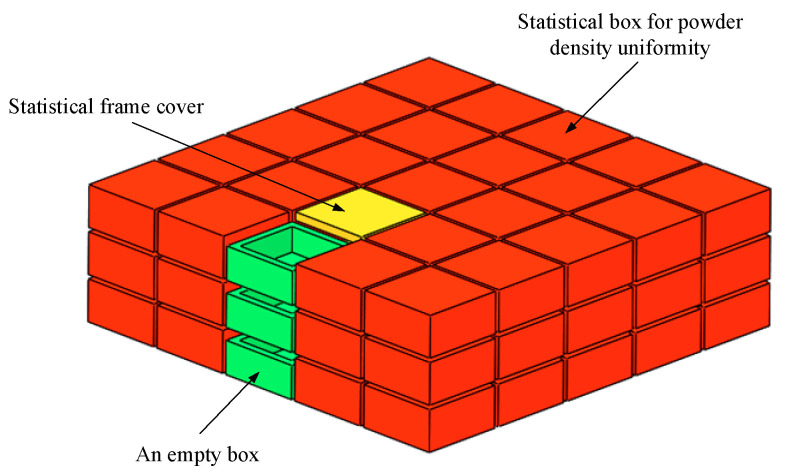
Sketch of the powder quality inspection design in the formation area.

In order to explore the influence of the powder laying parameters on the SLS powder laying quality and to verify the optimized test results, an experimental method of online sampling was designed to measure the powder laying quality. Figure 7 shows the schematic diagram of the SLS powder laying quality detection process. In the formation area, three experimental package layers can be seen, with each layer containing a powder paving roller working from the bottom up to 100 mm/s, 140 mm/s, and 227 mm/s, respectively. Each layer of the experimental package has the same design, including 13 statistical picker boxes, 1 no-cover statistical picker box, and 1 statistical picker box cover. The size of the outer cavity of the selection box is 20 mm × 20 mm × 10 mm, and the thickness of the cavity wall is 2 mm. The lumen is filled with powder. After sintering, it is cooled for a period of time and then the sintering package is removed. The sintered parts of the statistical selection box are then cleaned and sandblasted. The statistical selection boxes in each layer after cleaning are numbered and distinguished. A high-precision balance (accurate to 0.0001 g) is used to measure the mass $m_{pi}$ of each statistical selection box in each layer. Here, $m_{pi}$ can be expressed as:

(27) $$m_{pi}=m_{1i}-m_{2}-m_{3}$$

where $m_{2}$ is the mass of an open statistical box and $m_{3}$ is the mass of the statistical box cover.

The powder’s apparent density $\rho_{pi}^{\prime }$ in each statistical selection box is:

(28) $$\rho_{pi}^{\prime }=\frac{m_{pi}}{l_{i}w_{i}h_{i}}$$

where $l_{i}$, $w_{i}$, and $h_{i}$ represent the length, width, and height of the sintered parts in the statistical selection box, respectively.

The apparent density of the powder layer in the formation area is:

(29) $$\rho_{p}=\frac{\sum_{i=1}^{13}m_{pi}}{\sum_{i=1}^{13}l_{i}w_{i}h_{i}}$$

The standard deviation of laminar density in the formation zone is:

(30) $$S_{p}=\sqrt{\frac{1}{N-1}\sum_{i=1}^{N}{\left(\rho_{pi}^{\prime }-\bar{\rho_{pi}^{\prime }}\right)}^{2}}$$

where $\bar{\rho_{pi}^{\prime }}$ is the average value of the apparent density of the powder in the selection box.

**Figure 8:**
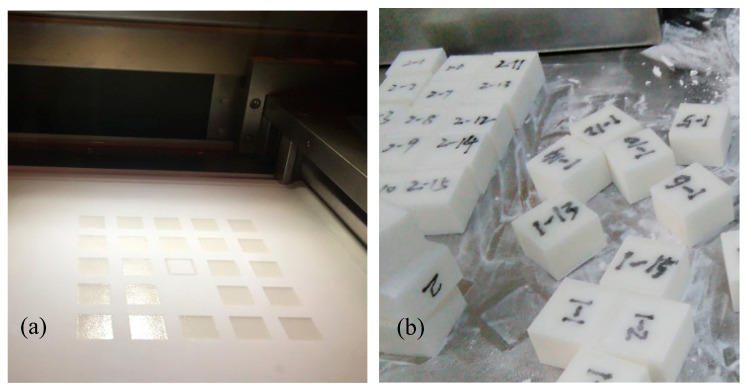
Statistical sintering experiment: (**a**) SLS molding process for the statistical box; (**b**) powder cleaning of sintered parts in the statistics box.

**Figure 9:**
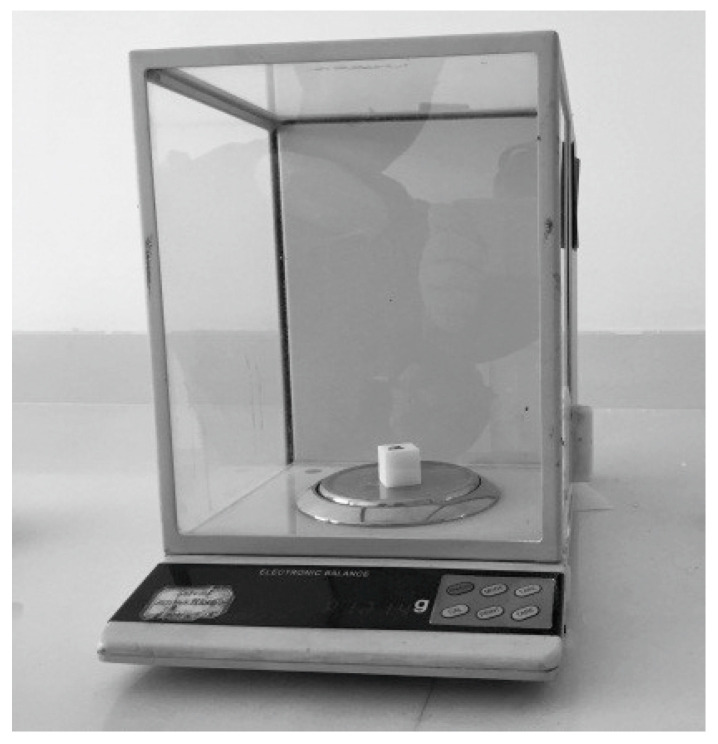
The size and quality parameters of sintered parts in the selection box.

According to the above experimental methods, the statistical box was prepared, as shown in Figure 8a. We selected the box to sinter the molded parts for powder cleaning (see Figure 8b). After cooling for a period of time, the size and quality parameters of the sintered parts in the statistical selection box were measured, as shown in Figure 9. 

**Figure 10:**
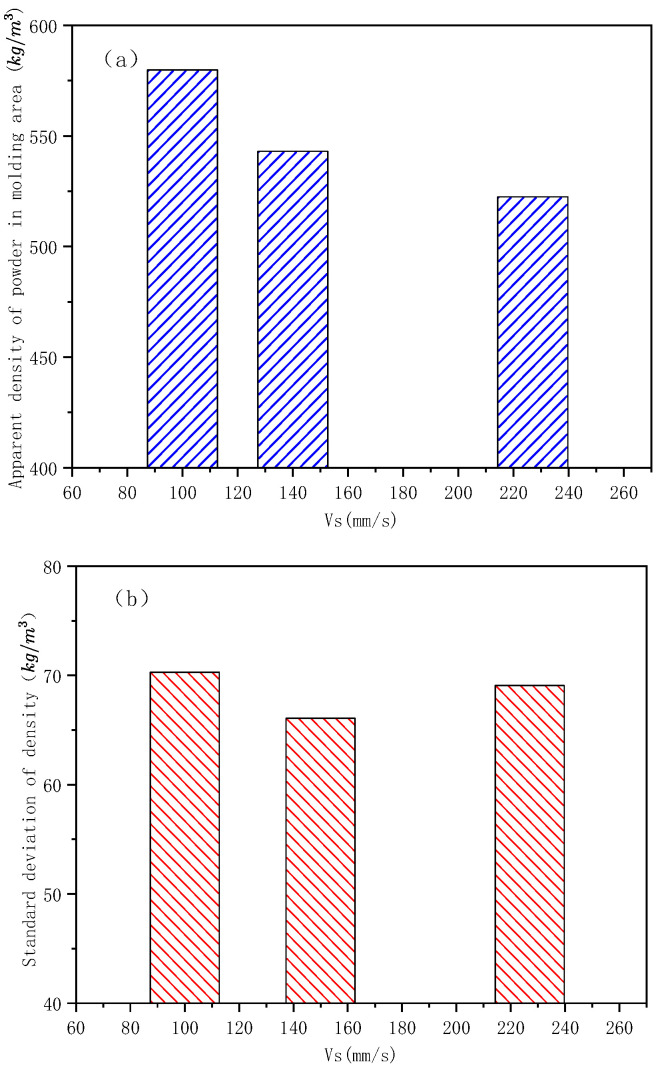
Experimental results of the powder spreading quality in the formation area (*V_r_*/*V_s_* = 0.5, *R_g_* = 20 mm, D50 = 50 μm): (**a**) effect of *V_s_* on the apparent density of powder in the formation zone; (**b**) effect of *V_s_* on the standard deviation of the formation zone density.

With the increase in drum speed, the apparent density of the powder in the formation area decreases (Figure 10). The reliability of the numerical simulation study on SLS powder laying process of nylon powder was verified. When the PA3200 powder D50 is 50 μm, the diameter of powder spreading drum is 40 mm, the ratio of the linear velocity to translation velocity of the drum is 0.5, the translation velocity of drum is 100 mm/s, the apparent powder density in the formation area is 579.8 $\mathrm{kg}/m^{3}$ (Figure 10a), and the standard deviation of the apparent powder density in the formation area is 70.3 $\mathrm{kg}/m^{3}$ (Figure 10b). This is in good agreement with the absolute value of optimization target result no. 1 in Table 5, and the errors are 2.38% and 1.69%, respectively. When the roller translation speed is 140 mm/s, the apparent powder density in the formation area is 543.1 $\mathrm{kg}/m^{3}$ and the standard deviation of the apparent powder density in the formation area is 66.1 $\mathrm{kg}/m^{3}$. This is in good agreement with the absolute value of optimization target result no. 14 in Table 5, and the errors are 2.82% and 4.59%, respectively. This shows that the experimental method of online sampling and measurement of the powder laying quality is feasible and that the multi-objective optimization results of the nylon powder laying quality in the SLS process based on the genetic algorithm are reliable.

## 5. Conclusions

The SLS powder spreading process was numerically simulated based on the DEM. The effects of the powder’s physical properties and operating conditions on the bed quality were investigated, characterized by the density characteristics, density uniformity, and flatness of the powder layer. The main results from the present study are summarized as follows:

(1) Statistical analysis and curve fitting of the DEM simulation data from the powder laying process were conducted based on the central composite experimental design method. ANOVA was used to modify the fitting model. A regression model of the powdering quality was established based on the RSM. The relationship between the proposed powdering quality index and the research variables was expressed well;
(2) An improved multi-objective optimization algorithm based on NSGA-II was used to optimize the powder laying quality of nylon powder in the SLS. The solutions in the optimized Pareto solution set were evenly distributed in the target space. An optimal compromise solution can be selected from Pareto optimal solution set according to the product requirements;
(3) The apparent density and standard deviation of the powder under different conditions were determined experimentally. The translation speed of the roller has a great influence on the powder laying quality, and the apparent powder density in the formation area decreases with the increase in roller speed. The experimental results agreed well with the selected optimization results and the maximum error was less than 4.6%. The reliability of the numerical simulation study on the SLS powder laying process of nylon powder was verified.

At present, it is difficult to accurately measure the force and deformation of the particle contacts using experimental equipment, and the inexact mechanical parameters are not conducive to modeling simulations and for improvement of the adhesion collision model. In addition, on the basis of improving the measurement method used for the particle electrostatic transfer characteristics, the particle band charge and electrification mechanism in this model need to be further refined. The model simulation system is smaller than the actual system, so the parallel calculation of the DEM may increase the simulation system and improve the computational efficiency.

## Figures and Tables

**Table 1:** DEM model parameters of PA3200 powder spreading process.

Parameter	Value
Density (kg/m^3^)	1000
Shear modulus of powder (MPa)	61
Poisson ratio of power	0.35
Wall density (kg/m^3^)	7800
Wall shear modulus (Gpa)	80
Poisson ratio of wall	0.30
Coefficient of sliding friction between powder and wall	0.51
Coefficient of rolling friction between powder and wall	0.15
Hamaker constant between powder and wall	9.72 × 10^−20^
Resilience factor between powder and wall	0.52
Coefficient of sliding friction between powders	0.48
Rolling friction coefficient between powder and wall surface	0.24
Springback coefficient between powders	0.11
Hamaker constant between powders (J)	7.21 × 10^−20^
Powder charge generation factor	0.03
Power D50 (μm)	50
Number of powder particles	215,000

**Table 2:** Working parameters of numerical simulation of powder laying process.

Parameter	Value
Drum translational velocity *Vs* (mm/s)	60, 100, 140, 180, 220, 260, 280, 320
Ratio of drum linear velocity to translational velocity *Vr*/*Vs*	0.16, 0.33, 0.50, 0.66, 1.0, 1.31, 2.0, 2.63
Diameter of roller *Rg* (mm)	4, 12, 20, 24, 28, 32, 36, 40
Powder particle D50 diameter (μm)	30, 40, 50, 60, 70, 80, 90, 100

**Table 3:** Design factor level of DEM simulation test for nylon powder laying process.

Test Factor	−1.414	−1	0	1	1.414
Drum translational velocity *Vs* (mm/s)	68.93	100.00	175.00	250.00	281.07
particle diameter d (μm)	39.46	50.00	75.00	100.00	110.36

**Table 4:** DEM simulation test scheme and simulation results of powder laying process (*Rg* = 20 mm, *Vr*/*Vs* = 0.5).

Test No.	Translational Velocity *Vs* (mm/s)	Particle Size D (μm)	Apparent Density (kg/m^3^)	Standard Deviation of the Density (kg/m^3^)	Roughness (μm)
1	175.00	75.00	535.00	79.60	42.04
2	100.00	100.00	542.10	113.70	40.60
3	175.00	75.00	535.00	79.60	42.04
4	175.00	39.64	572.20	75.60	43.02
5	250.00	100.00	549.40	124.90	44.21
6	250.00	50.00	558.40	82.50	43.12
7	175.00	75.00	535.00	79.60	42.04
8	175.00	110.36	557.70	133.90	42.14
9	281.07	75.00	535.80	95.10	45.25
10	68.93	75.00	553.30	90.80	44.36
11	100.00	50.00	563.50	67.30	44.37
12	175.00	75.00	535.00	79.60	42.04
13	175.00	75.00	535.00	79.60	42.04

**Table 5:** Partial Pareto optimal solution for multi-objective optimization of powdering quality via DEM simulation.

Test No.	x(1)	x(2)	f(1)	f(2)	f(3)
1	100.000	50.000	−566.332	71.509	44.637
2	145.201	52.547	−555.438	69.179	43.021
3	124.124	100.000	−545.108	113.938	41.099
4	153.701	55.058	−551.242	69.371	42.750
5	122.961	50.120	−562.044	69.813	43.759
6	105.078	50.003	−565.348	71.035	44.424
7	126.765	97.187	−542.746	108.696	41.219
8	151.930	68.351	−539.267	74.025	42.255
9	109.506	50.645	−563.553	70.626	44.202
10	129.048	88.767	−538.274	95.260	41.578
11	120.959	99.525	−544.970	113.299	41.123
12	118.713	98.379	−544.272	111.400	41.182
13	146.365	91.364	−538.048	98.421	41.471
14	139.111	50.677	−558.840	69.282	43.257
15	114.194	87.017	−539.307	93.891	41.798
16	124.878	95.907	−542.035	106.622	41.277
17	104.755	100.000	−546.880	115.748	41.181
18	133.703	50.750	−559.483	69.377	43.396
19	139.166	90.231	−538.007	96.921	41.494
20	152.929	66.994	−540.043	73.241	42.296

**Table 6:** SLS process parameters used in the experiment.

Parameter	Value
Laser power (W)	21
Scanning interval (mm)	0.15
Drum diameter (mm)	40
Ratio of drum linear velocity to translational velocity	0.5
Preheating temperature of formation cylinder (°C)	171
Preheating temperature of powder feeding cylinder (°C)	132

## Data Availability

The data presented in this study are available on request from the corresponding author.
